# Identifying barriers to lipoprotein(a) testing in secondary cardiovascular prevention and co-designing implementation strategies: an Italian behavior change wheel-informed study

**DOI:** 10.3389/fcvm.2026.1906713

**Published:** 2026-09-09

**Authors:** Marcello Arca, Rossella Marcucci, Aldo Pietro Maggioni, Claudio Bilato, Eugenio Stabile, Francesco Cipollone, Davide Capodanno, Giovanna Liuzzo, Ferdinando Varbella, Federico Guerra, Stefano Carugo, Diletta Valsecchi, Monica Cangini, Alessio Maria Ghiringhelli, Simone Parretti, Francesca Donnaloja, Alberico Luigi Catapano

**Affiliations:** 1 Department of Translational and Precision Medicine, Sapienza University of Rome, Rome, Italy; 2 Department of Experimental and Clinical Medicine, University of Florence, Florence, Italy; 3 ANMCO Research Center-Heart Care Foundation, Florence, Italy; 4 Division of Cardiology, West Vicenza General Hospitals, Arzignano, Italy; 5 Division of Cardiology, Department of Health Sciences, Università Della Basilicata, Potenza, Italy; 6 Clinica Medica Unit, Department of Medicine and Aging Sciences, University of Chieti-Pescara, Chieti, Italy; 7 Division of Cardiology, Azienda Ospedaliero-Universitaria Policlinico “G. Rodolico-San Marco”, University of Catania, Catania, Italy; 8 Department of Cardiovascular Sciences, Fondazione Policlinico Gemelli IRCCS, Rome, Italy; 9 Interventional Cardiology Unit, San Luigi Gonzaga University Hospital, Orbassano, Italy; 10 Infermi Hospital ASL TO3, Rivoli, Turin, Italy; 11 Cardiology and Arrhythmology Clinic, Department of Biomedical Sciences and Public Health, Marche Polytechnic University, Ancona, Italy; 12 Department of Clinical Sciences and Community Health, University of Milan and Fondazione Ospedale Maggiore IRCCS Policlinico Di Milano, Milan, Italy; 13 Novartis Farma S.p.A., Milan, Italy; 14 IQVIA Solutions Italy S.r.l, Milan, Italy; 15 Department of Pharmacological and Biomolecular Sciences, University of Milan, and IRCCS Multimedica, Milan, Italy

**Keywords:** behavior change wheel, COM-B model, implementation science, Italy, lipoprotein(a), secondary cardiovascular prevention

## Abstract

**Background:**

Lipoprotein(a) [Lp(a)] is a causal risk factor for atherosclerotic cardiovascular disease, which remains unaddressed by current lipid-lowering therapies. Given its established role as a genetic independent cardiovascular risk factor, national and international guidelines recommend screening for Lp(a) levels, as elevated concentrations indicate a higher risk of cardiovascular events. Despite these implications and the availability of a simple standardized assay reimbursed by the Italian National Health Service, Lp(a) testing remains largely underused in the Italian clinical practice. The aim of this project was to identify barriers to Lp(a) testing in clinical practice and to develop targeted strategies to promote its incorporation into secondary cardiovascular prevention strategies in Italy, using an implementation science framework.

**Methods:**

This project adopted a multi-method approach, using the Behavior Change Wheel (BCW) framework to identify barriers to Lp(a) testing. Barriers identified in the literature were mapped using the Capability, Opportunity, Motivation– Behavior (COM-B) model, and further characterized through the Theoretical Domains Framework. Each identified barrier was subsequently discussed and rated for perceived impact during a multidisciplinary working group involving 12 stakeholders (cardiologists, internists, lipidologists and laboratory professionals). Building on these findings, several intervention strategies were developed in line with BCW guidance and prioritized by stakeholders during a second multidisciplinary working group to ensure overall feasibility**.**

**Results:**

Capability-related barriers, including limited knowledge of Lp(a) as a cardiovascular risk factor, and uncertainty about its role on patient management, were among the most influential. Opportunity barriers included the absence of approved Lp(a)-lowering treatments, and a lack of test standardization. Motivational barriers included a lack of evidence of clinical benefit of Lp(a) reduction and limited awareness of its role in patient management. To address the identified barriers, training initiatives, creation of working groups, sharing of practical tools, alignment between clinicians and laboratories, and patient support emerged as high-priority interventions.

**Conclusion:**

To promote Lp(a) testing in secondary cardiovascular prevention Italian clinical practice, this project identified behavioral barriers across all COM-B domains and designed actionable key strategies using a theory-driven approach. Future research should pilot these activities to overcome identified barriers and improve patient management.

## 1 Introduction

Lipoprotein(a) [Lp(a)] is a plasma low-density lipoprotein (LDL)–like particle, characterized by the presence of an apolipoprotein(a) covalently bound to apolipoprotein B-100 (1). Lp(a) plasma levels are largely genetically determined. Current guidelines suggest that elevated levels of Lp(a) (>50 mg/dL or >125 nmol/L), values observed in approximately 20% of the global population (2–4), are associated with an increased risk of atherosclerotic cardiovascular disease, even among individuals with well-controlled LDL cholesterol levels. Furthermore, genetic cohort studies have demonstrated that elevated Lp(a) is an independent causal risk factor for coronary heart disease, myocardial infarction, and aortic valve stenosis (4–7).

Given its prognostic relevance, the European Atherosclerosis Society (EAS) and the European Society of Cardiology (ESC) recommend at least one lifetime measurement of Lp(a) in all adults to identify individuals with high levels and refine cardiovascular risk estimation (8). Further recommendations include the incorporation of Lp(a) testing into secondary cardiovascular prevention pathways (4). Indeed, beyond its established role in primary prevention, elevated Lp(a) is strongly associated with recurrent cardiovascular events in secondary prevention settings (4, 9–11). Increasing Lp(a) plasma levels are correlated with progressively higher incidence rates of major adverse cardiovascular events (4, 7, 10), underscoring the pivotal role of Lp(a) as a biomarker for risk stratification in secondary prevention (12, 13).

Although strongly recommended by international guidelines and requiring only a standard, non-fasting blood draw (14), Lp(a) testing remains largely underused in Italian clinical practice (15). To address this implementation gap in Italian clinical practice, this project applied the Behavior Change Wheel (BCW) framework to identify multilevel barriers to Lp(a) testing in secondary cardiovascular prevention within the Italian hospital care setting and develop tailored strategies that support the integration of Lp(a) screening into routine care.

## 2 Methods

### 2.1 Context analysis

A narrative literature review was conducted to explore current practices, barriers, and facilitators related to Lp(a) testing, with a focus on the Italian healthcare system and its organizational and clinical context. Bibliographic search was conducted in PubMed following PRISMA guidelines and supplemented by snowballing (see Supplementary Material). In addition, given the implementation-oriented and Italian-focused scope of the project, grey literature sources were reviewed to capture organizational, policy, and reimbursement-related evidence relevant to the Italian healthcare context that may not be adequately represented in bibliographic databases. Sources included clinical guidelines, consensus statements, scientific society publications, and reimbursement-related materials. Relevant documents were identified through targeted searches of institutional and scientific society websites and through reference screening of publications retrieved from the bibliographic search. No restrictions on publication date were applied, and only articles published in English and Italian were considered.

### 2.2 Framework identification for behavioral analysis

Based on barriers identified in the literature, a predominantly behavioral challenge emerged, prompting the adoption of the BCW framework to guide project design. The BCW provides a structured, systematic, multistep methodology for examining different types of determinants, including but not limited to, behavioral ones, and developing implementation strategies (16). Briefly, the process begins by defining the problem, selecting target behaviors, and specifying actors and context. Next, behaviors that need to change are identified using the Capability, Opportunity, Motivation–Behavior (COM-B) model, complemented by the Theoretical Domains Framework (TDF), which expands COM-B into 14 domains for a more granular analysis (17). This combined approach enables systematic mapping of barriers and facilitators. Subsequent steps involve selecting intervention functions to address identified barriers and translating interventions into operational actions through behavior change techniques. Finally, delivery modes are defined to implement the interventions (16).

### 2.3 Behavioral diagnosis

A Multistakeholder Working Group (MWG) of 12 stakeholders was established to ensure contextual relevance and multidisciplinary engagement, based on their recognized expertise in either the management of secondary cardiovascular prevention or Lp(a) testing. Stakeholder involvement was restricted to the hospital care setting, given evidence suggesting that post-discharge follow-up and risk-factor monitoring after an acute cardiovascular event is mainly in charge of cardiologists, internists, lipidologists and may be suboptimal in the community setting in Italy (18, 19). The considered stakeholders including also laboratory professionals were identified as affiliated with different hospital centers across Italy, ensuring a balanced geographical representation and accounting for potential variability in clinical practice patterns.

The barriers preliminarily identified from the literature review were systematically mapped onto the COM-B domains and further elaborated using the TDF framework. The categorized barriers were subsequently presented to the stakeholders during the first MWG session to gather both qualitative and quantitative insights into the behavioral determinants influencing Lp(a) testing in clinical practice. Stakeholders were asked to rate the perceived impact of each barrier on a five-point Likert scale (from 1 = no impact on behavior, to 5 = very high impact on behavior). During the MWG, participants were also given the opportunity to propose additional barriers from their clinical experience and organizational perspectives. Participants then engaged in an open discussion, allowing a collective reflection on the relevance and completeness of the identified barriers, and reaching a shared understanding of which factors most strongly affected Lp(a) testing. This interactive process ensured that the final list of barriers accurately reflected both the published evidence and real-world challenges influencing Lp(a) testing in clinical practice.

The MWG workshop was transcribed verbatim and analyzed by two researchers independently. Transcript coding was guided by the predefined COM-B and TDF frameworks, and discrepancies between reviewers were resolved through consensus. The use of two independent reviewers and consensus-based resolution of discrepancies was intended to enhance consistency in construct assignment and minimize analytical bias. During the workshop, stakeholders were presented with each TDF construct and asked to assess its perceived impact on Lp(a) test prescribing in clinical practice using a five-point Likert scale. Ratings were collected through an online survey accessed via a QR code displayed during the meeting. The mean score across all TDF constructs was subsequently calculated and used as a reference threshold (3.67). Constructs with a mean stakeholder rating between 3.67 and <4 were initially assigned a relevance score of 2 (moderate relevance), whereas constructs with a mean rating ≥4 were initially assigned a relevance score of 3 (high relevance). Constructs with a mean rating below 3.67 were assigned a relevance score of either 1 (if between 3 and <3.67) or 0 (if ≤3). In parallel, qualitative analysis explored the extent to which each construct emerged during workshop discussions and captured the specific barriers that stakeholders associated with the different TDF constructs. To integrate quantitative and qualitative findings, a margin of ±0.3 around the reference threshold of 3.67 was applied. For constructs with mean ratings falling within this interval, the relevance score could be increased or decreased by one level based on the frequency and prominence with which the construct emerged during the workshop discussion. The final relevance score ranged from level 0 to 3 (0 = not relevant, 1 = low relevance, 2 = moderate relevance, and 3 = high relevance).

Following the integration of quantitative and qualitative findings, only barriers associated with constructs receiving final relevance scores of 2–3 were retained for reporting and subsequently used to inform the development of implementation strategies.

### 2.4 Action plan: development and prioritization of intervention strategies

Following the BCW framework, a set of potential intervention strategies was developed, based on behavior change techniques adapted to the specific context of the project and defined as actionable implementation strategies to facilitate behavior change (20).

Activity prioritization was carried out using selected APEASE criteria (Affordability, Practicability, Effectiveness/cost-effectiveness, Acceptability, Side-effects/safety, Equity) from the BCW framework (21). In particular, the dimensions of Affordability, Practicability, Effectiveness, and Acceptability were applied. Side-effects/safety and Equity were excluded because the implementation strategies under evaluation were primarily educational and organizational in nature, and no issues specifically related to these dimensions emerged during the assessment process. In addition, a further criterion of Scalability was introduced, recognizing that the ability to expand an intervention was essential to ensure long-term sustainability and maximize population-level impact (22).

**Figure 1:**
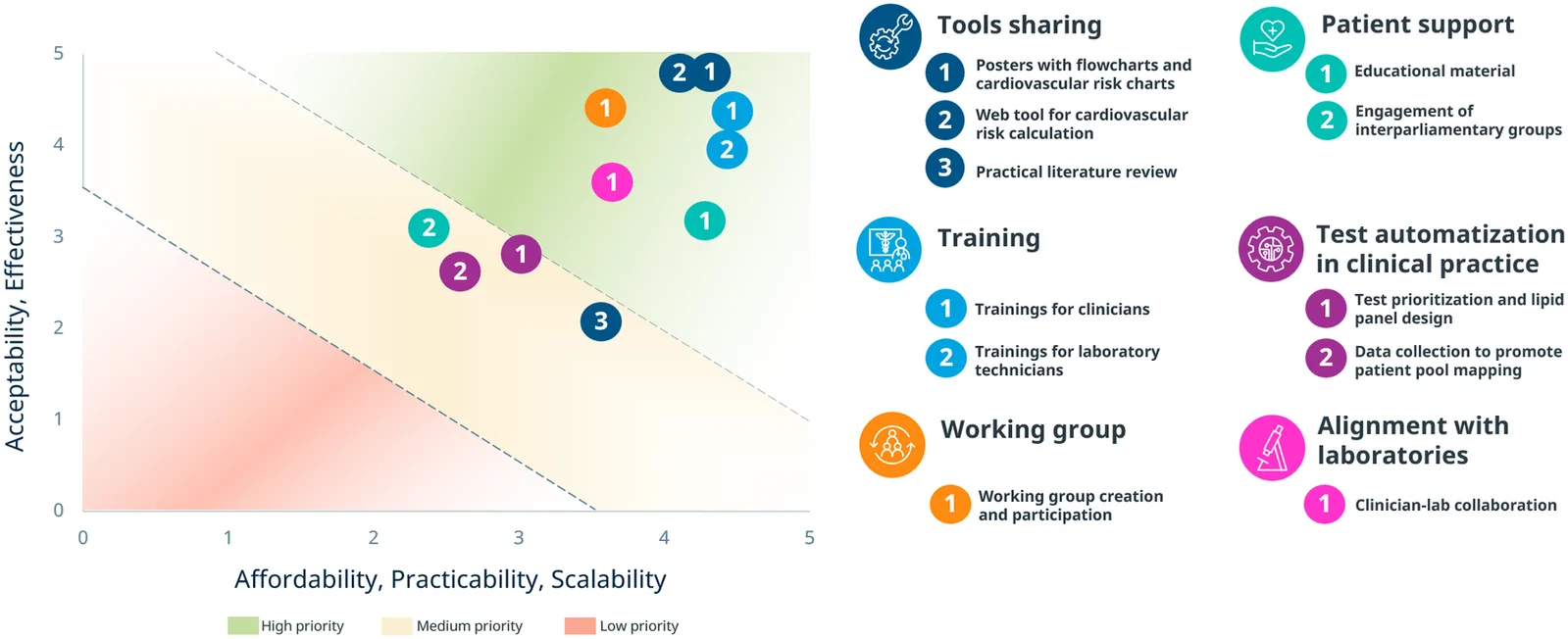
Prioritization of implementation strategies to increase Lp(a) testing in clinical practice. Each intervention was assessed according to two composite dimensions: Acceptability/Effectiveness (vertical axis) and Affordability, Practicability, and Scalability (horizontal axis). The background color gradient indicates overall priority level: green = high priority, yellow = medium priority, and red = low priority. Lp(a), Lipoprotein(a).

Each strategy was initially assessed by the research team in terms of practicability, scalability, and affordability—using a five-point Likert scale (1 = very low to 5 = very high)—to ensure a consistent evaluation across all proposed strategies. Subsequently, all strategies were presented and discussed during a second MWG session in which the 12 stakeholders evaluated them for acceptability and effectiveness, and rated them on a five-point Likert scale. Once all ratings had been collected, two mean scores were calculated: one combining acceptability and effectiveness, and the other combining practicability, scalability, and affordability. An overall mean score was then calculated as the average of these two composite scores, thereby assigning equal weight to the stakeholder-related (acceptability/effectiveness) and implementation-related (practicability/scalability/affordability) dimensions. Strategies with an overall average score ≥3 were classified as high priority. In the prioritization matrix (Figure 1), this threshold corresponds to the condition (x + y)/2 ≥ 3, where x and y represent the two composite scores, and is graphically represented by the upper diagonal boundary (x + y = 6). To enable visual prioritization, the proposed strategies were plotted on a scatterplot using their scores. High-priority strategies were co-designed with stakeholders to ensure contextual relevance and were refined into targeted, operational actions, thereby enhancing their feasibility and applicability within real-world clinical and organizational settings. Strategies that did not meet the high-priority threshold were excluded from further development. The set of refined, high-priority actions formed the final implementation toolkit, providing a practical and consensus-based framework to support the systematic adoption of Lp(a) testing in the Italian healthcare system.

**Figure 2:**
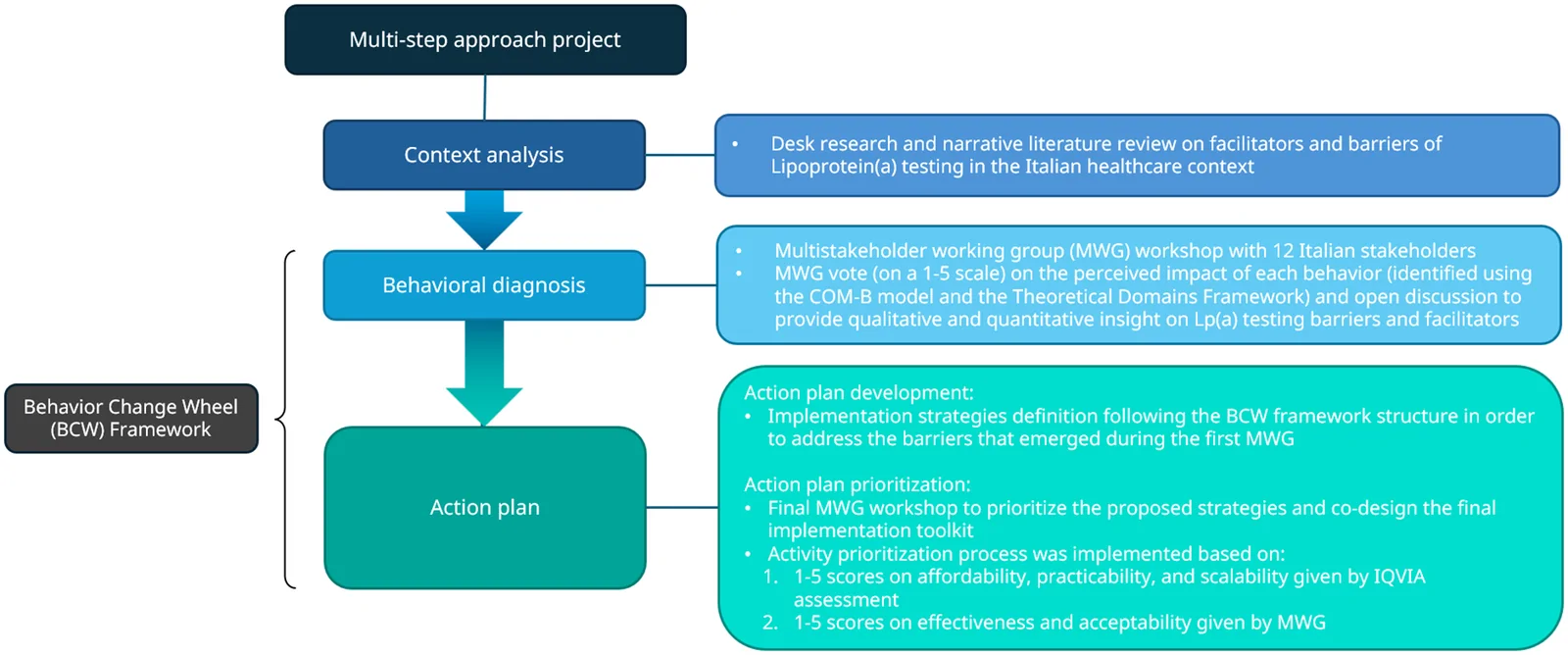
Project flowchart. BCW, Behavior Change Wheel; COM-B, Capability, Opportunity, Motivation–Behavior; Lp(a), Lipoprotein(a); MWG, Multistakeholder Working Group.

The overall methodological approach is summarized in Figure 2.

## 3 Results

### 3.1 Context analysis through narrative literature review

A focused literature review was conducted to identify barriers and facilitators related to Lp(a) testing in Italian clinical practice. The literature search retrieved a total of 9,839 records from PubMed, and, after removal of duplicates, the remaining articles were screened. Based on a review of titles and abstracts, 52 full-text articles were analyzed, and, through snowballing, 108 articles were included in the qualitative synthesis (see Supplementary Material). The evidence generated from the literature review suggested that the underuse of Lp(a) testing was primarily driven by behavioral barriers rather than technical or organizational issues (15).

### 3.2 Perceived impact of barriers in clinical practice: first MWG results

**Figure 3:**
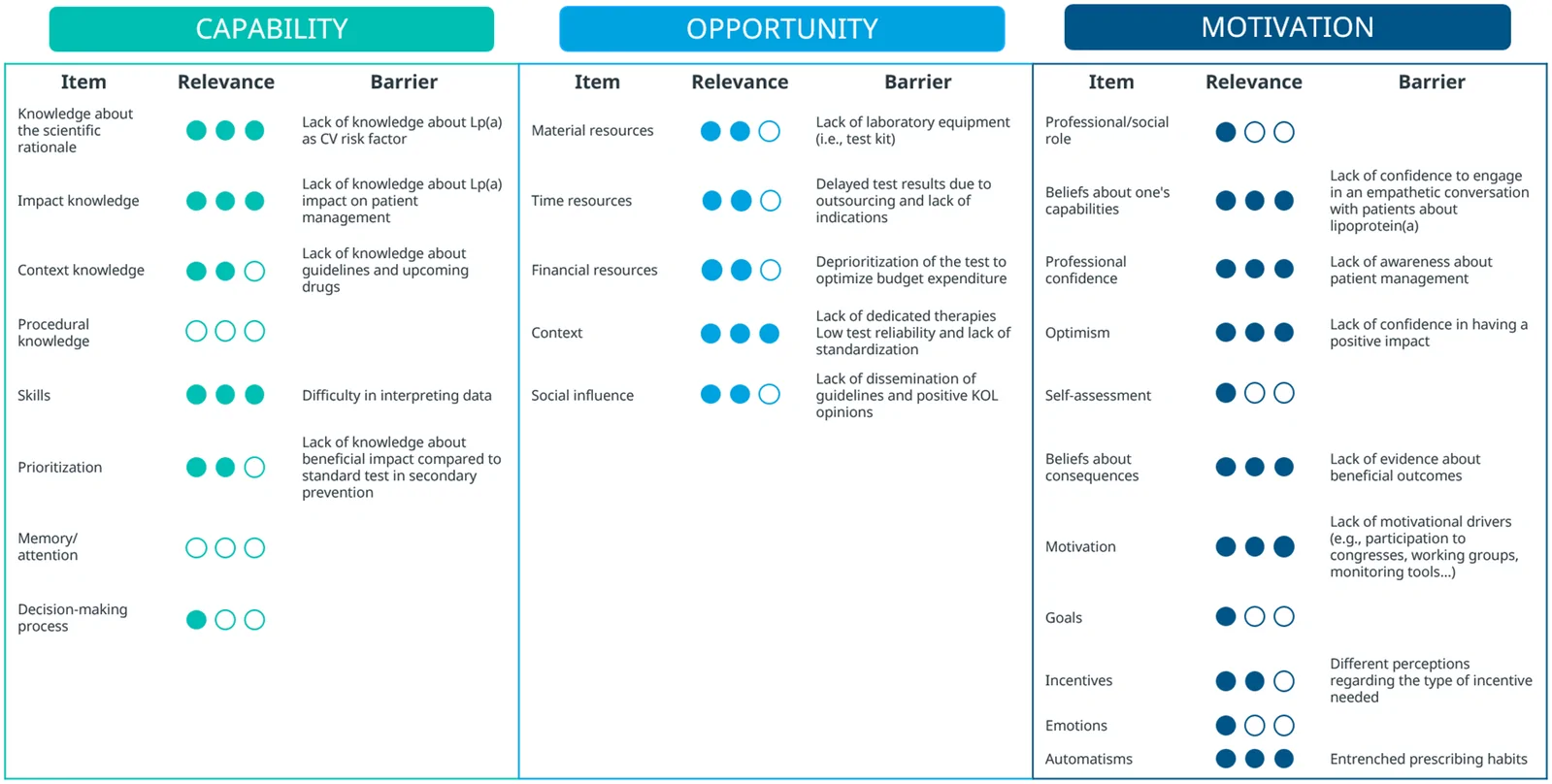
Assessment of TDF constructs and associated barriers influencing Lp(a) testing according to the COM-B model. Items represent TDF constructs mapped to the COM-B domains. Relevance scores reflect the integrated assessment of each construct based on stakeholder ratings and workshop discussion. Barriers are reported only for constructs rated as moderately or highly relevant (relevance scores 2-3). COM-B, Capability, Opportunity, Motivation–Behavior; CV, cardiovascular; KOL, Key Opinion Leader; Lp(a), Lipoprotein(a); TDF, Theoretical Domains Framework.

Twenty-four TDF constructs, mapped across the three domains of the COM-B framework (Capability, Opportunity, and Motivation), were discussed and rated by the 12 stakeholders during the first MWG workshop. Constructs receiving moderate or high relevance scores were subsequently explored qualitatively to identify the barriers reported in Figure 3.

Within the Capability domain, the TDF constructs Knowledge about the scientific rationale, Impact knowledge, and Skills achieved high relevance scores (relevance = 3). Qualitative analysis of the workshop discussions identified the lack of knowledge of Lp(a) as a cardiovascular risk factor, limited understanding of its impact on patient management, and difficulties in data interpretation as the main barriers associated with these constructs. Context knowledge and Prioritization were rated as moderately relevant (relevance = 2), with stakeholders highlighting limited knowledge of guidelines and emerging therapies, as well as uncertainty regarding the additional value of Lp(a) testing compared with standard assessments in secondary prevention.

Within the Opportunity domain, the construct Context achieved the highest relevance score (relevance = 3). Associated barriers included the absence of dedicated therapies, concerns regarding test reliability, and lack of assay standardization. Material resources, Time resources, Financial resources, and Social influence were rated as moderately relevant (relevance = 2), and were associated with barriers including limited laboratory equipment, delayed test results due to outsourcing, deprioritization of Lp(a) testing to optimize budget expenditure, and insufficient dissemination of guidelines and positive KOL opinions.

Within the Motivation domain, the constructs Professional confidence, Optimism, Beliefs about consequences, Motivation, and Automatism were rated as highly relevant (relevance = 3). Qualitative discussions linked these constructs to barriers such as uncertainty regarding patient management, lack of confidence in achieving a positive clinical impact, limited evidence of beneficial outcomes, lack of motivational drivers, and entrenched prescribing habits. Incentives were rated as moderately relevant (relevance = 2), with stakeholders reporting uncertainty regarding the most appropriate forms of incentive to support Lp(a) testing.

### 3.3 Activity prioritization: second MWG results

To overcome the specific challenges identified, eleven targeted interventions, grouped into six overarching categories (training, tools sharing, working group, patient support, test automatization in clinical practice, and alignment with laboratories), were developed using the BCW and rated for affordability, practicability, and scalability by the researchers and for acceptability and effectiveness by the 12 stakeholders during the second MWG. The overall results are summarized in Figure 1. The interventions rated as high priority were within five of the six categories identified (i.e., tools sharing, training, working group, patient support, and alignment with laboratories).

Tool-sharing initiatives, including the development of posters featuring flowcharts and cardiovascular risk charts, as well as a web-based tool based on a dedicated algorithm integrating multiple clinical variables to estimate individual cardiovascular risk, were regarded as strategic priorities, with very high acceptability and effectiveness. Training programs targeting both clinicians and laboratory professionals were considered essential to address the lack of knowledge regarding Lp(a) as a cardiovascular risk factor, its implications for patient management, and challenges in data interpretation. These programs received high ratings for affordability, practicability, and scalability, with good scores for acceptability and effectiveness. The creation and participation in working groups also achieved elevated ratings across the five criteria, while closer clinician-lab collaboration and the production and distribution of educational material to patients were associated with slightly lower scores for affordability, practicability, and scalability, though maintaining good acceptability and effectiveness. All high-priority interventions were further refined and thoroughly co-designed with stakeholders to ensure that the resulting actions were highly targeted and actionable. The final selected actions, along with corresponding barriers and intervention areas, are summarized in Table 1.

**Table 1:** Mapping of identified barriers to corresponding implementation strategies and activities for Lp(a) testing.

Barriers addressed	Area of action	Implementation activity
• Lack of knowledge about Lp(a) as a cardiovascular risk factor • Lack of knowledge about Lp(a) impact on patient management • Difficulty in interpreting data • Lack of dedicated therapies • Lack of laboratory equipment (i.e., test kit) • Deprioritization of the test to optimize budget expenditure	Training	1. Development of interactive recorded training for both clinicians and laboratory professionals
• Lack of knowledge about Lp(a) as a cardiovascular risk factor • Lack of knowledge about Lp(a) impact on patient management • Lack of knowledge about guidelines and upcoming drugs • Difficulty in interpreting data • Lack of dissemination of guidelines and positive KOLs' opinions • Lack of awareness about patient management	Tools sharing	1. Promotion of a web algorithm-based cardiovascular risk calculator 2. Design of posters showing cardiovascular risk charts and patient management flowcharts
• Lack of confidence in having a positive impact • Lack of awareness about patient management • Lack of self-monitoring tools • Lack of motivational drivers	Working group	1. Creation of a working group coordinated by a KOL and open to all HCPs in the pilot centers
• Low test reliability and lack of standardization • Delayed test results due to outsourcing and lack of indications	Alignment with laboratories	1. Test management, prescription and report optimization through clinician-lab collaboration
• Lack of confidence to engage in an empathetic conversation with patients about Lp(a)	Patient support	1. Development and promotion of educational material to be handed out during visits

HCPs, healthcare professionals; KOL, key opinion leader; Lp(a), lipoprotein(a).

Actions rated as medium priority included a practical literature review to synthesize and disseminate key information on Lp(a), test prioritization and lipid panel design, patient data collection for pool mapping, and engagement of interparliamentary groups to foster institutional collaboration on cardiovascular risk prevention. Because these activities were classified as medium priority, they were not further discussed or incorporated into the implementation toolkit.

## 4 Discussion

Despite the well-established prognostic relevance of Lp(a), clear guideline recommendations advocating at least one lifetime measurement, and recent evidence suggesting that repeat testing may provide additional clinical information in selected circumstances, the uptake of Lp(a) testing in Italian clinical practice remains low (15, 23). To address this gap, this project is, to our knowledge, the first in Italy to systematically examine barriers to prescribing Lp(a) testing in secondary cardiovascular prevention, using a structured and validated methodological framework.

A comprehensive review of the literature has identified several behavioral barriers to Lp(a) testing, suggesting that improving Lp(a) implementation requires interventions targeting behavior and decision-making processes (15). In light of these findings, and informed by studies employing comparable methodological approaches in different thematic contexts such as self-care and adherence in heart failure, cardiac rehabilitation and statin use (24–27), we adopted the BCW (16) as a guiding framework to structure our intervention strategy. The active involvement of stakeholders in our project strengthened the process, supporting contextual relevance and consensus across clinical domains.

Consistent with previous international studies in cardiovascular prevention (13, 28), capability-related determinants such as knowledge and awareness gaps emerged as important factors limiting test adoption. Despite limitations in representation, involved stakeholders identified limited knowledge of Lp(a) as a cardiovascular risk factor and uncertainty regarding its implications for patient management as relevant barriers. These findings confirm gaps in familiarity with current guideline recommendations and the role of Lp(a) in secondary cardiovascular prevention, despite growing evidence supporting its value as a prognostic marker (4, 29). To overcome these capability-related barriers, a set of targeted and high-priority actions was delineated and co-designed during the second MWG, focusing on strategies to enhance clinicians' knowledge and awareness. Dissemination of practical tools (algorithm-based cardiovascular risk calculators and management flowcharts), establishment of working groups to foster knowledge-sharing among clinicians, and specific training for both clinicians and laboratory personnel were among the suggested actions. Indeed, awareness initiatives have been shown to markedly improve Lp(a) testing. In the PATRIOT-QI project, targeted education led to a 24-fold increase in Lp(a) screening, enabling earlier detection of high-risk individuals and optimization of preventive management (27).

Opportunity-related barriers that emerged in our project, such as the lack of specific Lp(a) targeting therapies and concerns about low test reliability, highlighted the need to improve clinicians' awareness of the clinical utility of Lp(a) testing, as supported by the growing scientific consensus on its clinical relevance and by guideline recommendations for universal screening (30, 31). Indeed, although targeted Lp(a)-lowering therapies are currently under development (32), and not yet available in clinical practice, Lp(a) measurement is clinically valuable for refining cardiovascular risk stratification and identifying patients who may benefit from more intensive preventive management. In the absence of specific treatments, current management focuses on risk stratification and intensive control of other modifiable cardiovascular risk factors, including cholesterol, glucose, weight, diet, physical activity, smoking, and blood pressure (4, 7, 33). Lipoprotein apheresis remains the only intervention currently capable of significantly reducing Lp(a) concentrations, although its effect is transient and requires repeated treatment sessions (4, 7, 34). The concerns about low test reliability identified in our project appear to be closely linked to persistent challenges in assay standardization and result interpretation. Our findings confirmed that a lack of assay standardization and inconsistent interpretation of results hinder adoption, consistent with previous reports showing that these barriers persisted even among informed clinicians (4, 35). Indeed, multiple assay types remain in use, with values reported in mg/dL and nmol/L that are not directly interchangeable, leading to misestimation and limiting comparability across results (28, 36, 37). To address these barriers, we propose training initiatives that can play a pivotal role in promoting Lp(a) testing for accurate cardiovascular risk stratification and supporting optimal patient management. Moreover, we recommend interventions aimed at strengthening internal collaboration between clinicians and laboratories, establishing shared testing protocols, and ensuring consistency in reporting, strategies also supported by international recommendations for long-term implementation success (38–40).

Beyond the implementation strategies proposed in our project, greater awareness of existing contextual facilitators within the Italian healthcare system may further support the adoption of Lp(a) testing. These include the simplicity of the assay, its relatively low cost (€4.85 according to the Italian Ministry of Health) (48), and reimbursement by the National Health Service.

Motivational factors and patient-facing components also require careful consideration. Our analysis highlighted that clinicians perceived challenges in communicating elevated, genetically determined Lp(a) values to patients, particularly in the absence of effective Lp(a) lowering drug therapies. In this context, clinician–patient interaction should focus on providing accurate information and reassurance. Therefore, we proposed the development of patient support brochures designed to raise awareness of Lp(a) as a cardiovascular risk factor and to support clinician-patient communication regarding the role and implications of Lp(a) testing.

Through a comprehensive analysis of barriers and a collaborative co-design process with stakeholders, this project has delivered a key outcome: an implementation toolkit designed to facilitate the integration of Lp(a) testing into Italian secondary cardiovascular prevention pathways, and prioritized for broad applicability and potential scalability across healthcare settings.

### 4.1 Strengths and limitations

A key strength of this project is the integration of behavioral theory with stakeholder engagement, enabling the identification of context-sensitive solutions that reflect real-world, clinical and organizational challenges within the Italian healthcare system. Furthermore, the combined use of quantitative ratings and qualitative stakeholder discussions provided complementary perspectives to inform barrier assessment and the prioritization of implementation strategies.

Nonetheless, some limitations should be acknowledged. First, the prioritization process relied on the perspectives of a limited number of stakeholders involved in secondary cardiovascular prevention. Although participants were selected to provide multidisciplinary expertise and geographical distribution, the findings may only partially reflect the views of all healthcare professionals involved in Lp(a) testing and patient management across different care settings. Second, the absence of a representative of a Patient Advocacy Group (PAG) in the MWG may represent a limitation of the study. The involvement of PAGs should be considered in future research, particularly given that patient engagement emerged as a barrier in clinical practice and patient support was identified as a potential solution in the final implementation toolkit. Notably, patient-related barriers and the importance of clinician-patient communication were not predefined areas of investigation but emerged during stakeholder discussions and the subsequent behavioral analysis. Finally, as with most BCW applications, the identification and prioritization of barriers and implementation strategies were based on stakeholder perceptions and expert judgment rather than on direct observation of clinical behaviors or the evaluation of implementation outcomes.

## 5 Conclusion

Using a theory-driven approach, this project identified and prioritized several implementation strategies to support the prescription of Lp(a) testing in clinical practice. Tools sharing and training interventions achieved the highest prioritization scores. Other highly prioritized strategies included the establishment of working groups, strengthening collaboration between clinicians and laboratory personnel, and patient support through targeted educational materials. Future research is needed to pilot these prioritized strategies in real-world clinical practice and evaluate their feasibility, implementation, and impact on clinician behavior and Lp(a) testing uptake. Although this project focused on secondary cardiovascular prevention within the hospital care setting, several of the identified barriers are likely relevant beyond this context, including primary prevention settings. Future research should therefore explore the applicability and relevance of the prioritized strategies across different healthcare settings and patient populations.

Taken together, the implementation strategies identified and prioritized in this study address both behavioral and structural determinants of Lp(a) testing and may support its adoption in Italian clinical practice, with the potential to enhance cardiovascular risk stratification and improve patient management.

## Data Availability

The original contributions presented in the study are included in the article/Supplementary Material, further inquiries can be directed to the corresponding author.

## References

[1] Nordestgaard BG Langsted A. Lipoprotein(a) and cardiovascular disease. Lancet. (2024) 404(10459):1255–64. 10.1016/S0140-6736(24)01308-4 39278229

[2] Nordestgaard BG Chapman MJ Ray K Borén J Andreotti F Watts GF. Lipoprotein(a) as a cardiovascular risk factor: current status. Eur Heart J. (2010) 31(23):2844–53. 10.1093/eurheartj/ehq386 20965889 PMC3295201

[3] Patel AP Wang M Pirruccello JP Ellinor PT Ng K Kathiresan S. Lp(a) [lipoprotein(a)] concentrations and incident atherosclerotic cardiovascular disease: new insights from a large national biobank. Arterioscler Thromb Vasc Biol. (2021) 41(1):465–74. 10.1161/ATVBAHA.120.315291 33115266 PMC7769893

[4] Kronenberg F Mora S Stroes ESG Ference BA Arsenault BJ Berglund L. Lipoprotein(a) in atherosclerotic cardiovascular disease and aortic stenosis: a European atherosclerosis society consensus statement. Eur Heart J. (2022) 43(39):3925–46. 10.1093/eurheartj/ehac361 36036785 PMC9639807

[5] Grundy SM Stone NJ Bailey AL Beam C Birtcher KK Blumenthal RS. 2018 AHA/ACC/AACVPR/AAPA/ABC/ACPM/ADA/AGS/APhA/ASPC/NLA/PCNA guideline on the management of blood cholesterol: a report of the American College of Cardiology/American Heart Association task force on clinical practice guidelines. Circulation. (2019) 139(25):e1082–e143. 10.1161/CIR.0000000000000625 30586774 PMC7403606

[6] Pearson GJ Thanassoulis G Anderson TJ Barry AR Couture P Dayan N. 2021 Canadian cardiovascular society guidelines for the management of dyslipidemia for the prevention of cardiovascular disease in adults. Can J Cardiol. (2021) 37(8):1129–50. 10.1016/j.cjca.2021.03.016 33781847

[7] Chiesa G Zenti MG Baragetti A Barbagallo CM Borghi C Colivicchi F. Consensus document on lipoprotein(a) from the Italian society for the study of atherosclerosis (SISA). Nutr Metab Cardiovasc Dis. (2023) 33(10):1866–77. 10.1016/j.numecd.2023.07.019 37586921

[8] Mach F Baigent C Catapano AL Koskinas KC Casula M Badimon L. 2019 ESC/EAS guidelines for the management of dyslipidaemias: lipid modification to reduce cardiovascular risk. Eur Heart J. (2020) 41(1):111–88. 10.1093/eurheartj/ehz455 31504418

[9] Colantonio LD Bittner V Safford MM Marcovina S Brown TM Jackson EA. Lipoprotein(a) and the risk for coronary heart disease and ischemic stroke events among black and white adults with cardiovascular disease. J Am Heart Assoc. (2022) 11(11):e025397. 10.1161/JAHA.121.025397 35621195 PMC9238745

[10] Madsen CM Kamstrup PR Langsted A Varbo A Nordestgaard BG. Lipoprotein(a)-lowering by 50 mg/dL (105 nmol/L) may be needed to reduce cardiovascular disease 20% in secondary prevention: a population-based study. Arterioscler Thromb Vasc Biol. (2020) 40(1):255–66. 10.1161/ATVBAHA.119.312951 31578080

[11] MacDougall DE Tybjærg-Hansen A Knowles JW Stern TP Hartsuff BK McGowan MP. Lipoprotein(a) and recurrent atherosclerotic cardiovascular events: the US family heart database. Eur Heart J. (2025) 46(44):4762–75. 10.1093/eurheartj/ehaf297 40331569 PMC12634116

[12] Gianfagna F Poli S Costanzo S Di Castelnuovo A Panzera T De Curtis A. Lipoprotein(a) as an early marker of cardiovascular events in high-risk subjects: insights from the moli-sani cohort study. Front Cardiovasc Med. (2025) 12:1571395. 10.3389/fcvm.2025.1571395 40697997 PMC12279821

[13] Kronenberg F Bedlington N Ademi Z Geantă M Silberzahn T Rijken M. The Brussels international declaration on lipoprotein(a). Testing and Management. Atherosclerosis. (2025) 406:119218. 10.1016/j.atherosclerosis.2025.119218 40340180

[14] Ciffone N McNeal CJ McGowan MP Ferdinand KC. Lipoprotein(a): an important piece of the ASCVD risk factor puzzle across diverse populations. Am Heart J Plus. (2024) 38:100350. 10.1016/j.ahjo.2023.100350 38510747 PMC10945898

[15] Olmastroni E Casula M Xie S Catapano AL. Atherosclerotic cardiovascular disease and measurement of lipoprotein(a) levels in Italy. Eur J Atheroscler. (2024) 3(3):67–72. 10.56095/eaj.v3i3.77

[16] Michie S Van Stralen MM West R. The behaviour change wheel: a new method for characterising and designing behaviour change interventions. Implement Sci. (2011) 6:42. 10.1186/1748-5908-6-42 21513547 PMC3096582

[17] Atkins L Francis J Islam R O'Connor D Patey A Ivers N. A guide to using the theoretical domains framework of behaviour change to investigate implementation problems. Implement Sci. (2017) 12(1):77. 10.1186/s13012-017-0605-9 28637486 PMC5480145

[18] Gargiulo G Esposito G. Opportunità cliniche e impatto sul sistema sanitario di un trattamento ottimale del paziente post-sindrome coronarica acuta. Glob Reg Health Technol Assess. (2022) 9(Suppl 1):17–26. 10.33393/grhta.2022.2391 36628067 PMC9796606

[19] Lettieri C Colombo P Rosiello R Morici N Parogni P Musumeci G. A novel standard protocol of long-term follow-up shared with general practitioners after percutaneous coronary intervention: appropriateness and economic impact. G Ital Cardiol (Rome). (2015) 16(10):565–73. 10.1714/2028.22042 26444215

[20] Michie S Richardson M Johnston M Abraham C Francis J Hardeman W. The behavior change technique taxonomy (v1) of 93 hierarchically clustered techniques: building an international consensus for the reporting of behavior change interventions. Ann Behav Med. (2013) 46(1):81–95. 10.1007/s12160-013-9486-6 23512568

[21] Michie S Atkins L West R. The Behaviour Change Wheel: A Guide to Designing Interventions. London: Silverback Publishing (2014).

[22] Milat A Newson R King L Rissel C Wolfenden L Bauman A. A guide to scaling up population health interventions. Public Health Res Pract. (2016) 26(1):e2611604. 10.17061/phrp2611604 26863167

[23] Harb T Ziogos E Blumenthal RS Gerstenblith G Leucker TM. Intra-individual variability in lipoprotein(a): the value of a repeat measure for reclassifying individuals at intermediate risk. Eur Heart J Open. (2024) 4(5):oeae064. 10.1093/ehjopen/oeae064 39219855 PMC11365507

[24] Herber OR Atkins L Stork S Wilm S. Enhancing self-care adherence in patients with heart failure: a study protocol for developing a theory-based behaviour change intervention using the COM-B behaviour model (ACHIEVE study). BMJ Open. (2018) 8(9):e025907. 10.1136/bmjopen-2018-025907 30206096 PMC6144404

[25] Hoppchen I Wurhofer D Meschtscherjakov A Smeddinck JD Kulnik ST. Targeting behavioral factors with digital health and shared decision-making to promote cardiac rehabilitation-a narrative review. Front Digit Health. (2024) 6:1324544. 10.3389/fdgth.2024.1324544 38463944 PMC10920294

[26] Sanchez A Elizondo-Alzola U Pijoan JI Mediavilla MM Pablo S Sainz de Rozas R. Applying the behavior change wheel to design de-implementation strategies to reduce low-value statin prescription in primary prevention of cardiovascular disease in primary care. Front Med (Lausanne). (2022) 9:967887. 10.3389/fmed.2022.967887 36314033 PMC9606599

[27] Radfar N Ponn J Singh H Siddiqui E Shah M Fanous M. Promoting lipoprotein(a) awareness and testing for risk identification through outreach and teaching (PATRIOT-QI). J Clin Lipidol. (2025) 19(3):e6–7. 10.1016/j.jacl.2025.04.010

[28] Virani SS Koschinsky ML Maher L Mehta A Orringer CE Santos RD. Global think tank on the clinical considerations and management of lipoprotein(a): the top questions and answers regarding what clinicians need to know. Prog Cardiovasc Dis. (2022) 73:32–40. 10.1016/j.pcad.2022.01.002 35063437

[29] Amaritei O Mierlan OL Gutu C Gurau G. Lipoprotein(a): assessing the current knowledge and gaps in screening and treatment-A. Narrative Review. J Cardiovasc Dev Dis. (2025) 12(5):169. 10.3390/jcdd12050169 PMC12112544 40422940

[30] Catapano AL Daccord M Damato E Humphries SE Neely RDG Nordestgaard BG. How should public health recommendations address lp(a) measurement, a causative risk factor for cardiovascular disease (CVD)? Atherosclerosis. (2022) 349:136–43. 10.1016/j.atherosclerosis.2022.02.013 35292153

[31] Reyes-Soffer G Yeang C Michos ED Boatwright W Ballantyne CM. High lipoprotein(a): actionable strategies for risk assessment and mitigation. Am J Prev Cardiol. (2024) 18:100651. 10.1016/j.ajpc.2024.100651 38646021 PMC11031736

[32] Greco A Finocchiaro S Spagnolo M Faro DC Mauro MS Raffo C. Lipoprotein(a) as a pharmacological target: premises, promises, and prospects. Circulation. (2025) 151(6):400–15. 10.1161/CIRCULATIONAHA.124.069210 39928714

[33] Kronenberg F Mora S Stroes ESG Ference BA Arsenault BJ Berglund L. Frequent questions and responses on the 2022 lipoprotein(a) consensus statement of the European atherosclerosis society. Atherosclerosis. (2023) 374:107–20. 10.1016/j.atherosclerosis.2023.04.012 37188555

[34] Nugent AK Gray JV Gorby LK Moriarty MP. Lipoprotein apheresis: first FDA indicated treatment for elevated lipoprotein(a). Journal of Clinical Cardiology. (2020) 1(1):16–21. 10.33696/cardiology.1.002

[35] Kotani K. Consideration of possible involvement of oxidation of lp(a) in the association between lp(a) and atrial fibrillation. Eur J Prev Cardiol. (2025). 10.1093/eurjpc/zwaf531 40838972

[36] Shah NP Pajidipati NJ McGarrah RW Navar AM Vemulapalli S Blazing MA. Lipoprotein (a): an update on a marker of residual risk and associated clinical manifestations. Am J Cardiol. (2020) 126:94–102. 10.1016/j.amjcard.2020.03.043 32336532 PMC7376542

[37] Marcovina SM Albers JJ. Lipoprotein (a) measurements for clinical application. J Lipid Res. (2016) 57(4):526–37. 10.1194/jlr.R061648 26637278 PMC4808779

[38] Cegla J France M Marcovina SM Neely RDG. Lp(a): when and how to measure it. Ann Clin Biochem. (2021) 58(1):16–21. 10.1177/0004563220968473 33040574 PMC7791270

[39] Bhatia HS Boccara F. Lipoprotein(a): don't Forget about secondary prevention. Eur J Prev Cardiol. (2024) 31(15):1888–9. 10.1093/eurjpc/zwae276 39166475

[40] Catapano AL Perrone Filardi P Oliva F Agnello L Baragetti A Berteotti M. Lipoproteina(a) e rischio cardiovascolare: una roadmap per la gestione del paziente. Documento congiunto della società italiana per lo studio dell'Aterosclerosi (SISA), della società italiana di cardiologia (SIC), della associazione nazionale medici cardiologi ospedalieri (ANMCO) e di SIBioC. Biochim Clin. (2025) 49:278–96. 10.23736/S0393-0564.25.00077-9

