# Correction: Identifying barriers to lipoprotein(a) testing in secondary cardiovascular prevention and co-designing implementation strategies: an Italian behavior change wheel-informed study

**DOI:** 10.3389/fcvm.2026.1995870

**Published:** 2026-10-12

**Authors:** Marcello Arca, Rossella Marcucci, Aldo Pietro Maggioni, Claudio Bilato, Eugenio Stabile, Francesco Cipollone, Davide Capodanno, Giovanna Liuzzo, Ferdinando Varbella, Federico Guerra, Stefano Carugo, Diletta Valsecchi, Monica Cangini, Alessio Maria Ghiringhelli, Simone Parretti, Francesca Donnaloja, Alberico Luigi Catapano

**Affiliations:** 1 Department of Translational and Precision Medicine, Sapienza University of Rome, Rome, Italy; 2 Department of Experimental and Clinical Medicine, University of Florence, Florence, Italy; 3 ANMCO Research Center-Heart Care Foundation, Florence, Italy; 4 Division of Cardiology, West Vicenza General Hospitals, Arzignano, Italy; 5 Division of Cardiology, Department of Health Sciences, Università Della Basilicata, Potenza, Italy; 6 Clinica Medica Unit, Department of Medicine and Aging Sciences, University of Chieti-Pescara, Chieti, Italy; 7 Division of Cardiology, Azienda Ospedaliero-Universitaria Policlinico “G. Rodolico-San Marco”, University of Catania, Catania, Italy; 8 Department of Cardiovascular Sciences, Fondazione Policlinico Gemelli IRCCS, Rome, Italy; 9 Interventional Cardiology Unit, San Luigi Gonzaga University Hospital, Orbassano, Italy; 10 Infermi Hospital ASL TO3, Rivoli, Turin, Italy; 11 Cardiology and Arrhythmology Clinic, Department of Biomedical Sciences and Public Health, Marche Polytechnic University, Ancona, Italy; 12 Department of Clinical Sciences and Community Health, University of Milan and Fondazione Ospedale Maggiore IRCCS Policlinico Di Milano, Milan, Italy; 13 Novartis Farma S.p.A., Milan, Italy; 14 IQVIA Solutions Italy S.r.l, Milan, Italy; 15 Department of Pharmacological and Biomolecular Sciences, University of Milan, and IRCCS Multimedica, Milan, Italy

**Keywords:** behavior change wheel, COM-B model, implementation science, Italy, lipoprotein(a), secondary cardiovascular prevention

The author contributions statement was erroneously given as MA: Conceptualization, Writing – review & editing. RM: Conceptualization, Writing – review & editing. APM: Conceptualization, Writing – review & editing. CB: Conceptualization, Writing – review & editing. ES: Conceptualization, Writing – review & editing. FC: Conceptualization, Writing – review & editing. DC: Conceptualization, Writing – review & editing. GL: Conceptualization, Writing – review & editing. FV: Conceptualization, Writing – review & editing. FG: Conceptualization, Writing – review & editing. SC: Conceptualization, Writing – review & editing. DV: Writing – review & editing, Funding acquisition. MC: Funding acquisition, Writing – review & editing. AMG: Formal analysis, Writing – original draft, Writing – review & editing, Conceptualization. SP: Conceptualization, Formal analysis, Writing – original draft, Writing – review & editing. FD: Conceptualization, Formal analysis, Writing – original draft, Writing – review & editing. ALC: Conceptualization, Writing – review & editing.

The correct author contributions statement is MA: Study Design, Data Collection, Writing – review & editing. RM: Study Design, Data Collection, Writing – review & editing. APM: Study Design, Data Collection, Writing – review & editing. CB: Study Design, Data Collection, Writing – review & editing. ES: Study Design, Data Collection, Writing – review & editing. FC: Study Design, Data Collection, Writing – review & editing. DC: Study Design, Data Collection, Writing – review & editing. GL: Study Design, Data Collection, Writing – review & editing. FV: Study Design, Data Collection, Writing – review & editing. FG: Study Design, Data Collection, Writing – review & editing. SC: Study Design, Data Collection, Writing – review & editing. DV: Conceptualization and Study Design, Writing – review & editing. MC: Conceptualization and Study Design, Writing – review & editing. AMG: Conceptualization and Study Design, Data Collection, Formal Analysis, Writing – original draft, Writing – review & editing. SP: Conceptualization and Study Design, Data Collection, Formal Analysis, Writing – original draft, Writing – review & editing. FD: Conceptualization and Study Design, Data Collection, Formal Analysis, Writing – original draft, Writing – review & editing. ALC: Study Design, Data Collection, Writing – review & editing.

An incorrect Funding statement was provided. The Funding statement was erroneously given as “The author(s) declared that financial support was received for this work and/or its publication. This project was funded by Novartis Farma S.p.A. The funder was not involved in the study design, collection, analysis, interpretation of data, the writing of this article, or the decision to submit it for publication.” The correct funding statement reads “The author(s) declared that financial support was received for this work and/or its publication. This project was funded by Novartis Farma S.p.A., Italy.

The conflict of interest statement was erroneously given as “AMG, SP and FD have disclosed that they are employees of IQVIA Solutions SRL. DV and MC are employees of Novartis Farma S.p.A. MA received research funding from Amgen, Amryt, Chiesi, Daiichi Sankyo, Ionis Pharmaceutical, Lilly, Novartis, Pfizer, Regeneron, Sanofi, Ultragenyx, and honoraria for advisory boards, consultancy or speaker bureau from Amarin, Amgen, Amryt, Chiesi, Daiichi Sankyo, Ionis Pharmaceutical, Novartis, Regeneron, Sanofi, Ultragenyx, Viatris. RM received lecture fees from Amgen, Sanofi, Daiichi Sankyo, Novartis. APM received personal fees for the participation in committees of studies sponsored by Bayer, Sanofi, Novartis, AstraZeneca, outside the present work. CB received lectures’ honoraria and consulting fees from: Amgen, Sanofi, Novartis, Daiichi, PIAM, Neopharmed, Amarin, Menarini, Servier, DOC generici, Novo Nordisk, Lilly. GL reports receiving personal fees for scientific speaking and advisory activities from AstraZeneca, Boehringer Ingelheim, Novo Nordisk, Daiichi Sankyo, Sanofi, and Novartis. ALC, in the last three years, has received honoraria, lecture fees or research grants from: Akcea Therapeutics, Amarin, Amgen, Amryt Pharma, AstraZeneca, Daiichi Sankyo, Esperion, Ionis Pharmaceutical, Medscape Education, Menarini, Merck, Mylan, Novartis, Novo Nordisk, PeerVoice, Pfizer, Recordati, Regeneron, Sanofi, The Corpus, Viatris.

The remaining author(s) declared that this work was conducted in the absence of any commercial or financial relationships that could be construed as a potential conflict of interest.”

The correct conflict of interest statement is “AMG, SP and FD have disclosed that they are employees of IQVIA Solutions SRL. DV and MC have disclosed that they are employees of Novartis Farma S.p.A., Italy. MA received research funding from Amgen, Amryt, Chiesi, Daiichi Sankyo, Ionis Pharmaceutical, Lilly, Novartis, Pfizer, Regeneron, Sanofi, Ultragenyx, and honoraria for advisory boards, consultancy or speaker bureau from Amarin, Amgen, Amryt, Chiesi, Daiichi Sankyo, Ionis Pharmaceutical, Novartis, Regeneron, Sanofi, Ultragenyx, Viatris. RM received lecture fees from Amgen, Sanofi, Daiichi Sankyo, Novartis. APM received personal fees for the participation in committees of studies sponsored by Bayer, Sanofi, Novartis, AstraZeneca, outside the present work. CB received lectures’ honoraria and consulting fees from: Amgen, Sanofi, Novartis, Daiichi, PIAM, Neopharmed, Amarin, Menarini, Servier, DOC generici, Novo Nordisk, Lilly. GL reports receiving personal fees for scientific speaking and advisory activities from AstraZeneca, Boehringer Ingelheim, Novo Nordisk, Daiichi Sankyo, Sanofi, and Novartis. ALC, in the last three years, has received honoraria, lecture fees or research grants from: Akcea Therapeutics, Amarin, Amgen, Amryt Pharma, AstraZeneca, Daiichi Sankyo, Esperion, Ionis Pharmaceutical, Medscape Education, Menarini, Merck, Mylan, Novartis, Novo Nordisk, PeerVoice, Pfizer, Recordati, Regeneron, Sanofi, The Corpus, Viatris.

The authors declare that this study received funding from Novartis S.p.A., Italy. The funder was involved in the study design, interpretation of data, the writing of this article, and the decision to submit it for publication.”

The original version of this article has been updated.

