# Supplementary material for: Identifying barriers to lipoprotein(a) testing in secondary cardiovascular prevention and co-designing implementation strategies: an Italian behavior change wheel-informed study
Source: Front Cardiovasc Med. 2026 Sep 9;13:1906713. doi: 10.3389/fcvm.2026.1906713 (PMC13597708; doi:10.3389/fcvm.2026.1906713)
Supplement: Supplementary file 1 [file Datasheet1.docx]

Supplementary Material

# Supplementary Table 1. PubMed search strategy and search strings used to identify literature supporting the development of the project.

| **Search topic** | **PubMed search string** |
| --- | --- |
| ASCVD | ("lipoprotein(a)" OR "Lp(a)") AND  (ASCVD OR "atherosclerotic cardiovascular disease") |
| Clinical guidelines | ("lipoprotein(a)" OR "Lp(a)") AND (guideline OR guidelines) |
| Italian context | ("lipoprotein(a)" OR "Lp(a)") AND (Italy OR Italian) |
| Clinical management and monitoring | ("lipoprotein(a)" OR "Lp(a)") AND (management[Title/Abstract] OR monitoring) |
| Testing | ("lipoprotein(a)" OR "Lp(a)") AND (test OR testing) |
| Therapies | ("lipoprotein(a)" OR "Lp(a)") AND (therapy OR therapies) |
| Behaviour change wheel | ("lipoprotein(a)" OR "Lp(a)") AND ("Behavior Change Wheel" OR "Behaviour Change Wheel" OR BCW) |
| Costs and reimbursement | ("lipoprotein(a)" OR "Lp(a)") AND (cost OR reimbursement) AND (Italy OR Italian) |

## Supplementary Figure 1. PRISMA flow diagram of the literature search performed to support the development of the project

* search: ("lipoprotein(a)" OR "Lp(a)") AND (ASCVD OR "atherosclerotic cardiovascular disease"); ("lipoprotein(a)" OR "Lp(a)") AND (guideline OR guidelines); ("lipoprotein(a)" OR "Lp(a)") AND (Italy OR Italian); ("lipoprotein(a)" OR "Lp(a)") AND (management[Title/Abstract] OR monitoring); ("lipoprotein(a)" OR "Lp(a)") AND (test OR testing); ("lipoprotein(a)" OR "Lp(a)") AND (therapy OR therapies); ("lipoprotein(a)" OR "Lp(a)") AND (cost OR reimbursement) AND (Italy OR Italian). Performed on 18 January 2025
